# Increased BMP-Smad signaling does not affect net bone mass in long bones

**DOI:** 10.3389/fphys.2023.1145763

**Published:** 2023-03-30

**Authors:** Maiko Omi, Tejaswi Koneru, Yishan Lyu, Ai Haraguchi, Nobuhiro Kamiya, Yuji Mishina

**Affiliations:** ^1^ Department of Biologic and Materials Sciences and Prosthodontics, University of Michigan School of Dentistry, Ann Arbor, MI, United States; ^2^ Department of Budo and Sport Studies, Faculty of Budo and Sport Studies, Tenri University, Nara, Japan

**Keywords:** bone morphogenetic protein, BMPR1A, osteoblast lineage cells, bone formation, bone resorption

## Abstract

Bone morphogenetic proteins (BMPs) have been used for orthopedic and dental application due to their osteoinductive properties; however, substantial numbers of adverse reactions such as heterotopic bone formation, increased bone resorption and greater cancer risk have been reported. Since bone morphogenetic proteins signaling exerts pleiotropic effects on various tissues, it is crucial to understand tissue-specific and context-dependent functions of bone morphogenetic proteins. We previously reported that loss-of-function of bone morphogenetic proteins receptor type IA (BMPR1A) in osteoblasts leads to more bone mass in mice partly due to inhibition of bone resorption, indicating that bone morphogenetic protein signaling in osteoblasts promotes osteoclast function. On the other hand, hemizygous constitutively active (ca) mutations for BMPR1A (*caBmpr1a* ^*wt/+*^) in osteoblasts result in higher bone morphogenetic protein signaling activity and no overt skeletal changes in adult mice. Here, we further bred mice for heterozygous null for *Bmpr1a (Bmpr1a* ^*+/−*^) and homozygous mutations of *caBmpr1a* (*caBmpr1a* ^*+/+*^) crossed with *Osterix*-Cre transgenic mice to understand how differences in the levels of bone morphogenetic protein signaling activity specifically in osteoblasts contribute to bone phenotype. We found that *Bmpr1a* ^*+/−*^, *caBmpr1a* ^*wt/+*^ and *caBmpr1a* ^*+/+*^ mice at 3 months of age showed no overt bone phenotypes in tibiae compared to controls by micro-CT and histological analysis although BMP-Smad signaling is increased in both *caBmpr1a* ^*wt/+*^ and *caBmpr1a* ^*+/+*^ tibiae and decreased in the *Bmpr1a* ^*+/−*^ mice compared to controls. Gene expression analysis demonstrated that slightly higher levels of bone formation markers and resorption markers along with levels of bone morphogenetic protein-Smad signaling, however, there was no significant changes in TRAP positive cells in tibiae. These findings suggest that changes in bone morphogenetic protein signaling activity within differentiating osteoblasts does not affect net bone mass in the adult stage, providing insights into the concerns in the clinical setting such as high-dose and unexpected side effects of bone morphogenetic protein application.

## 1 Introduction

Bone morphogenetic proteins (BMPs) were first described in 1965 as potent bone inducers due to their activities to form ectopic bones when implanted subcutaneously (Urist, 1965). Preclinical studies have demonstrated BMPs’ osteoinductive properties, especially for BMP-2, BMP-7 at 100–300 ng/mL *in vitro* and around 12 mg/site for new bone formation *in vivo* (8 mL of 1.5 mg/mL of recombinant human BMP-2) (Sampath and Reddi, 1981; Wozney et al., 1988; Luyten et al., 1989; Wozney, 1992; FDA, 2002). The United States Food and Drug Administration (FDA) has approved BMP-2 and BMP-7 for clinical use in non-union fractures long bone open-fractures, spinal fusion, and alveolar ridge augmentation (Gupta and Khan, 2005; Garrison et al., 2007; White et al., 2007). Genetic studies of human disorders fibrodysplasia ossificans progressiva (Shore et al., 2006) and chondrodysplasia (Thomas et al., 1996) indicate the importance of BMP signaling in the skeleton.

BMPs belong to the transforming growth factor-β (TGF-β) gene superfamily (Massague, 1998; Kishigami and Mishina, 2005) and signal through transmembrane serine/threonine kinase receptors. Upon ligand binding, BMP type I and BMP II receptors form heteromultimers (Wrana et al., 1994), and a constitutively active type II receptor kinase phosphorylates a GS box (a short stretch of the glycine- and serine-rich domain next to the transmembrane domains) in the type I receptor kinase to activate its activity. Activated BMP type I receptor kinases phosphorylate their downstream targets, Smad1, Smad5, and Smad9 proteins, and then interact with Smad4 to translocate into the nucleus (Chen et al., 2004). A point mutation in the GS box, for example, Q233D for BMPR1A and Q207D for ACVR1, makes type 1 receptor kinase activity constitutively active, however, type II receptors are still required for active Smad signaling (Bagarova et al., 2013).

BMP receptor type IA (BMPR1A) is abundantly expressed in bone and is activated by BMP-2 and BMP-4 ligands. Conventional knockout of *Bmp2*, *Bmp4* and *Bmpr1a* in mice results in embryonic lethality during gastrulation, which is before bone development, because BMPs are critical for the development of key organs including the heart and brain (Mishina et al., 1995; Winnier et al., 1995; Zhang and Bradley, 1996; Mishina et al., 1999). We previously inactivated *Bmpr1a* in an mature osteoblast-specific manner using *Og2*-Cre mice (Mishina et al., 2002; Mishina et al., 2004) and *Col1a1*-Cre mice (Kamiya et al., 2008a; Kamiya et al., 2008b). We also reported osteoblast-specific disruption of *Acvr1a* (Kamiya et al., 2011). It is interesting that in many cases the mutant mice exhibit more bone volume than littermate controls with some exceptions (Mishina et al., 2004; Kamiya et al., 2008a; Kamiya et al., 2008b; Kamiya et al., 2011). In contrast, gain-of-function of *Bmpr1a* in osteoblasts did not alter bone mass (Kamiya et al., 2020). Taken together with the facts that disruption of *Bmp2* and augmentation of *Bmp4* mutant mice both reduced bone mass (Okamoto et al., 2006; Tsuji et al., 2006), the mechanism of BMP signaling in controlling bone mass can be complicated and is not straightforward (Lowery and Rosen, 2018).

Along with the clinical use of BMP-2, it has been emerged that its efficacy and complications may be actual concerns (Woo, 2012a; b; 2013), including bone resorption and osteolysis (Pradhan et al., 2006). In fact, a phase I randomized study showed that the healing of open tibial fractures was not significantly accelerated by a BMP-2 loaded absorbable collagen sponge (Aro et al., 2011) presumably due to the increase in bone resorption (Seeherman et al., 2010). Additionally, various complications have been documented after spinal surgeries (Mroz et al., 2010; Carragee et al., 2011; Dmitriev et al., 2011; Williams et al., 2011), including vertebral resorption and osteolysis (Pradhan et al., 2006).

Studies from mouse genetics have demonstrated that BMPs and their signaling have pleiotropic roles in the different types of cells in the skeletal system, including mesenchymal cells, chondrocytes, osteoblasts, osteoclasts, and osteocytes (Kamiya and Mishina, 2011). To understand the clinical outcomes from BMP therapy, it is critical to define the roles of BMP signaling in bones in a cell type-dependent manner. We are interested to differentiate the impacts of BMP signaling in the early to late osteoblasts at physiologic levels. It is of interest that augmented BMP signaling in bone cells would affect bone resorption and bone mass, leading to a new insight into the potential use of BMPs in a clinical setting. To supplement our previous gene disruption studies, we conditionally activated the BMP-Smad signaling through BMPR1A in mice (*caBmpr1a*) using *Col1-CreERT* to report that a small upregulation of BMP-Smad signaling in osteoblasts does not show overt bone phenotypes (Kamiya et al., 2008b; Kamiya et al., 2020). In this study, we used a *Osterix*-Cre (Rodda and McMahon, 2006) to avoid possible impacts of tamoxifen treatments on bone phenotype. We bred *caBmpr1a* hemizygous mice to generate homozygous mice for the *caBmpr1a* transgene to further increase BMP-Smad signaling activity in *Osterix*-expressing cells to investigate alterations in bone phenotypes.

## 2 Materials and methods

### 2.1 Animals

Generation of the null mice for *Bmpr1a* (B6; 129S7-*Bmpr1a* ^*tm1Bhr*^/Mmnc, available at MMRRC, #016131-UNC) was previously described (Mishina et al., 1995). The heterozygous null of *Bmpr1a* were crossed with mice carrying the Tet-off *Osterix*-*Cre* (Tg (Sp7-tTA,tetO-EGFP/cre)1Amc, available at Jax Mice, #006361) (Rodda and McMahon, 2006) to obtain *Bmpr1a* ^*+/−*^ *;Osx-Cre* and *Bmpr1a* ^*+/+*^ *;Osx-Cre* mice. Mice conditionally expressing a constitutively active form of *Bmpr1a* (*caBmpr1a*) (B6; 129S7-Tg (CAG-lacZ,-BMPR1A*,-EGFP)1Mis/Mmjax, available at Jax Mice, #012436) (Kamiya et al., 2008b; Komatsu et al., 2013), which has a mutation in Q233D, were bred with mice carrying *Osx*-*Cre* to generate *caBmpr1a* hemizygous (*caBmpr1a* ^*wt/+*^ *;Osx-Cre*) and homozygous (*caBmpr1a* ^*+/+*^ *;Osx-Cre*) mice. Resulting mice showed ligand-independent activation of BMP-Smad signaling after Cre recombination. Because *caBmpr1a* transgenic line was generated through random transgenesis and we have not identified the inserted region, we differentiated hemizygous mice from homozygous mice by genomic real-time quantitative PCR using a custom designed TaqMan primer set (Yang et al., 2021) and on some occasions, genotyping results are confirmed by breeding with wild type mice. Activation of Osterix-Cre during embryogenesis did not cause lethality or overt morphogenic changes; therefore, we decided not to suppress Cre activity during embryogenesis, and mice were kept on regular diet and never treated with Doxycycline. All mice were kept in a mixed background of 129S7 and C57BL6/J and housed in a 12 h light/dark cycle with *ad libitum* access to food and water. All mouse experiments in this manuscript were approved by the Institutional Animal Care and Use Committee (IACUC) at the University of Michigan, Ann Arbor, and were conducted accordance with ARRIVE guidelines.

### 2.2 Micro-computed tomography (micro-CT)

Tibiae were harvested from 3-month-old male mice and fixed with 4% paraformaldehyde. The samples were placed in a 19 mm diameter specimen holder and scanned over the entire length of the tibia using a micro-CT system (µCT100 Scanco Medical, Bassersdorf, Switzerland) with voxel size 10 μm, 70 kVp, 114 μA, 0.5 mm AL filter, and integration time 500 ms. A 0.5 mm region of trabecular compartment was analyzed immediately below the growth plate using a fixed global threshold of 26% (260 on a grayscale of 0–1,000, or 569 mg HA/ccm); and a 0.3 mm region of cortical compartment at the midpoint was analyzed using a fixed global threshold of 36% (360 on a grayscale of 0–1,000, or 864 mg HA/ccm). Trabecular bone volume fraction (BV/TV), trabecular thickness (Tb. Th), trabecular number (Tb. N), trabecular separation (Tb. Sp), cortical bone volume fraction (BV/TV), cortical porosity, cortical thickness, bone mineral density (BMD), tissue mineral density (TMD), sub-periosteal area and sub-endosteal area were analyzed using an evaluation software from the manufacture.

### 2.3 Histology and histomorphometry

Samples were decalcified with 14% EDTA and a series of paraffin bone sections was made at 5 μm followed by hematoxylin and eosin (H&E) staining. For TRAP staining, decalcified samples were embedded in OCT to make 10 μm sections and stained with TRAP solution containing Naphthol AS-BI phosphoric acid, 2.5 M acetate buffer, 0.67 M tartrate solution. We used tibial sections for static histomorphometry. These measurements were made in a blinded, non-biased manner using ImageJ (Egan et al., 2012). The secondary spongiosa restricted to a square area 200 µm distal to the growth plate of the proximal tibia were used as regions of interest (ROIs). We followed the Report of the American Society of Bone and Mineral Research Histomorphometry Nomenclature Committee (Dempster et al., 2013) for measurements.

### 2.4 Immunohistochemistry

Tibiae were decalcified with 14% EDTA for 2 weeks before paraffin embedding. Deparaffinized sections were treated with 0.01 M citric acid (pH 6.0) for 20 min for antigen retrieval. The sections were treated with 3% hydrogen peroxide and blocking solution, then incubated with the primary phospho-Smad1/5/9 (pSmad1/5/9) antibody (Cell Signaling, cat # 13820, 1:100) at 4°C for 16 h. The sections were then reacted with HRP-conjugated goat anti-rabbit IgG (Abcam, cat # ab64241, no dilution). The ROIs were confined to the trabecular bone under the growth plate of the proximal tibia. The ratio of the number of pSmad1/5/9-positive cells to total cells located on the trabecular bone surface was quantified using ImageJ (Crowe and Yue, 2019).

### 2.5 Quantitative reverse transcription-polymerase chain reaction (qRT-PCR)

Bone marrow was flushed out from bones and the flushed tibia was used for RNA extraction using TRIzol reagent (Ambion). From 500 ng of RNA, cDNA was generated using SuperScript II cDNA Synthesis (Invitrogen). Gene expression levels were compared between different genotypes using Applied Biosystems ViiA7 platform. Endogenous GAPDH was used to normalize expression levels of each gene. The specificity of amplification was confirmed by checking melting curves. The primers for the SYBR Green quantification method are shown in Supplementary Table S1.

### 2.6 Cell culture and immunofluorescence staining

Bone marrow stromal cells (BMSCs) were isolated from bone marrows from the tibia of mice at 4 weeks old. Briefly, both ends of each tibia were cut, and bone marrow was flushed out by centrifugation. The collected bone marrow was cultured in 10% FBS/Dulbecco’s Modified Eagle Medium (DMEM) supplemented with antibiotics. BMSCs were seeded on glass coverslips in 24-well plates (5 × 10^4^ cells/well) and maintained in DMEM without FBS for 5 h. Cells were stimulated with 100 ng/mL of recombinant human BMP-2 (rhBMP-2, R&D, cat # 335-BM) for 30 min and then fixed in 4% paraformaldehyde for 20 min. Cells were sequentially incubated in 5% bovine serum albumin for 60 min and pSmad1/5/9 antibody at 4°C 16 h. Alexa Fluor 594 donkey anti-rabbit IgG (1:200, Invitrogen, cat # A32754) was used for fluorescent detection as a secondary antibody. Slides were mounted with ProLong Gold antifade reagent (Invitrogen, cat# P36934). The mean intensity for the red fluorescence per nuclei was measured using ImageJ (Shi et al., 2016).

### 2.7 Statistical analysis

Statistical analyses were done using one-way analysis of variance (ANOVA) among four groups and followed by a Tukey test. All experiments were done with three biological replicates or more per group. The results are expressed as the mean ± SD.

## 3 Results

### 3.1 Increase in BMP signaling activity in *caBmpr1a* mutant mice

To compare impacts of 4 different levels of BMP-Smad signaling in osteoblasts on adult long bone phenotypes, we set up breeding using conventional null allele for *Bmpr1a* and conditional constitutively activated *Bmpr1a* transgenic mouse line (*caBmpr1a*) to generate *Bmpr1a* ^*+/−*^, *Bmpr1a* ^*+/+*^ (wild type), *caBmpr1a* ^*wt/+*^ and *caBmpr1a* ^*+/+*^ mice, which we previously generated in our group (Mishina et al., 1995; Kamiya et al., 2008b; Komatsu et al., 2013). To achieve osteoblast-specific expression of *caBmpr1a*, we used *Osterix-Cre* mouse line of which Cre activity can be suppressed by Doxycycline for stage-specific genomic manipulation (Rodda and McMahon, 2006; Song et al., 2012). However, induction of *caBmpr1a* expression during embryogenesis did not lead to lethality or overt morphogenetic changes, we decided to keep breeding pairs and resulting pups on regular chow to maintain Cre activity throughout the experiments. To avoid misleading of the phenotypes that could be caused by presence of *Osterix-Cre*, but without Cre-dependent recombination (Razidlo et al., 2010; Davey et al., 2012; Wang et al., 2015), we selected mice carrying *Osterix-Cre* in 4 different genotypes of mice for comparisons.

**FIGURE 1:**
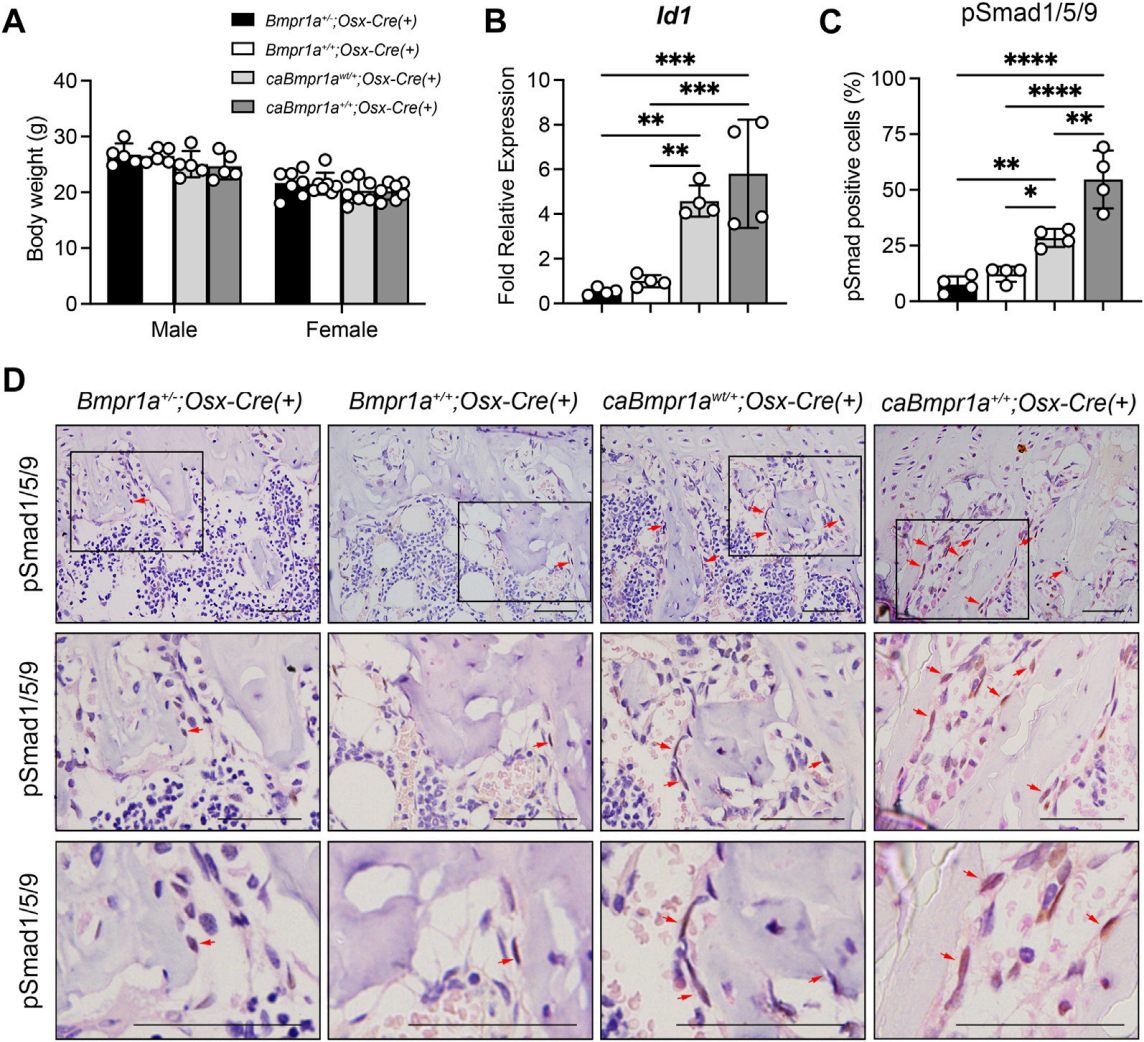
Genotype dependent upregulation of BMP-Smad signaling activity. **(A)** Body weight of the mice at 12 weeks of age (male: n = 5 for each group, female: n = 7 for each group). **(B)** Expression levels of *Id1*, a target gene of BMP signaling, in tibiae were measured at 12 weeks of age (n = 4 male for each group). **(C, D)** Immunohistochemical detection of phosphorylated form of Smad1/5/9 in tibiae at 12 weeks of age. pSmad1/5/9-positive cells (brown staining) were marked by red arrows. Higher magnification photos are also shown. Scale bar = 50 µm **(D)**. The ratio of pSmad1/5/9-positive cells (brown + blue) to total cells (blue) on the bone surface of the trabecular bone was analyzed (n = 4 male for each group) **(C)**. ****p < 0.0001, ***p < 0.001, **p < 0.01, and *p < 0.05.

At 12 weeks of age, the body weights of *Bmpr1a* ^*+/−*^ *;Osx-Cre*, *caBmpr1a* ^*wt/+*^ *;Osx-Cre* and *caBmpr1a* ^*+/+*^ *;Osx-Cre* mice were close to each other including controls (*Bmpr1a* ^*+/+*^ *;Osx-Cre*) in both sexes (Figure 1A). The levels of *Id1*, one of the direct targets of BMP-Smad signaling, upregulated in both *caBmpr1a* ^*wt/+*^ *;Osx-Cre* (4.6-fold) and *caBmpr1a* ^*+/+*^ *;Osx-Cre* (5.8-fold) tibiae, and downregulated in the *Bmpr1a* ^*+/−*^ *;Osx-Cre* (0.5-fold) at 3 months compared to controls (Figure 1B). For canonical BMP signaling, the levels of phosphorylated forms of Smad1/5/9 in osteoblast lineage cells (brown-stained cells at the bone surface) were significantly higher in both the trabecular bone of the *caBmpr1a* ^*wt/+*^ *;Osx-Cre* (2.3-fold) and *caBmpr1a* ^*+/+*^ *;Osx-Cre* (4.5-fold) tibiae and lower in the *Bmpr1a* ^*+/−*^ *;Osx-Cre* (0.6-fold) at 3 months compared to controls (Figures 1C, D).

### 3.2 No overt skeletal changes in *Bmpr1a* heterozygous null and *caBmpr1a* mutant mice

**FIGURE 2:**
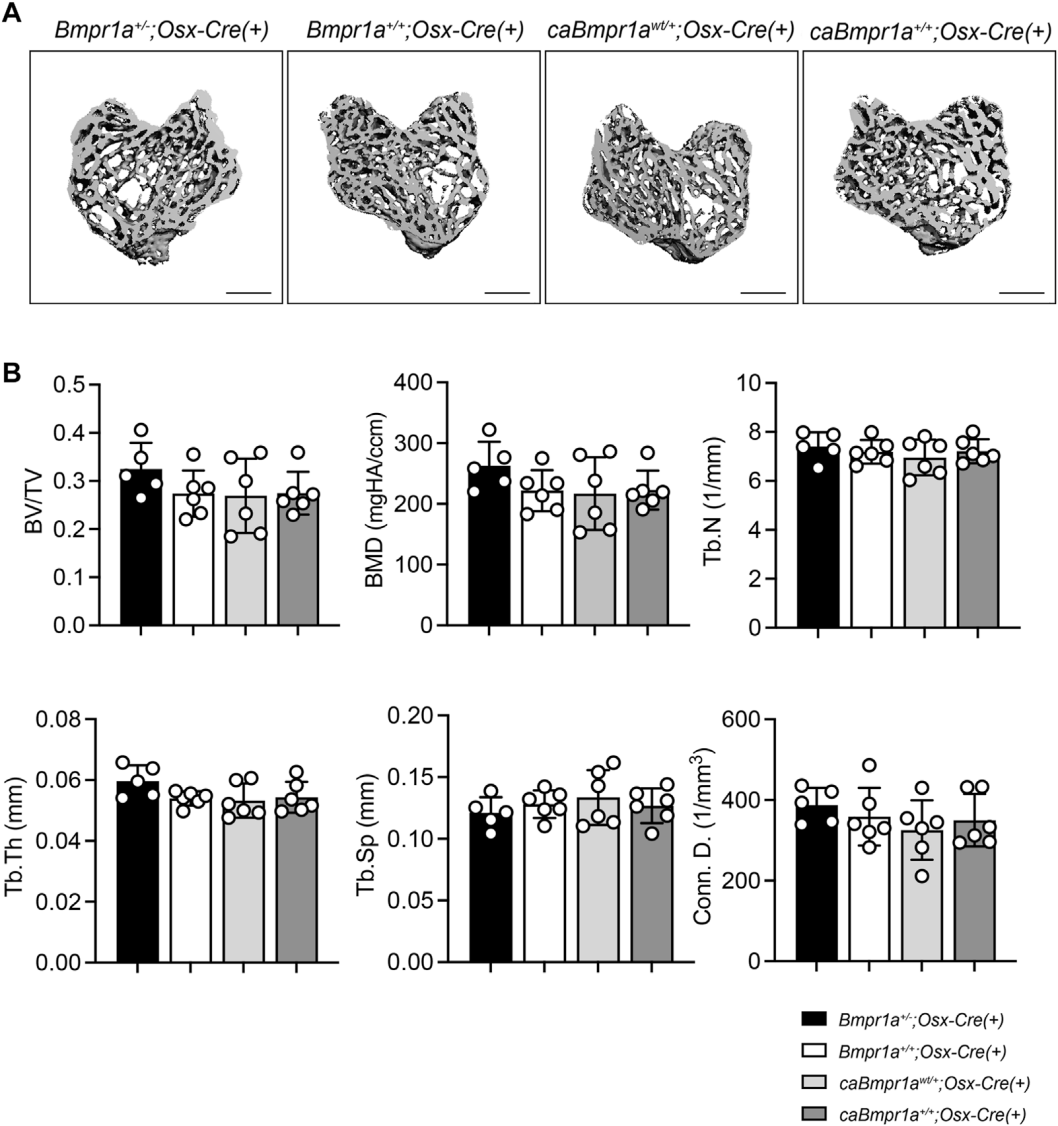
Micro-CT analysis for trabecular compartments of the male mouse tibia at 12 weeks of age. **(A)** Micro-CT based 3D images of the mouse proximal tibia. Scale bar = 500 µm. **(B)** Bone volume (BV/TV), bone mineral density (BMD), trabecular number (Tb.N), trabecular thickness (Tb.Th), trabecular space (Tb.Sp) and connective density (Conn.D.) were analyzed (n = 5 male for *Bmpr1a* ^*+/−*^ *;Osx-Cre* mice, n = 6 male for *Bmpr1a* ^*+/+*^ *;Osx-Cre, caBmpr1a* ^*wt/+*^ *;Osx-Cre, caBmpr1a* ^*+/+*^ *;Osx-Cre* mice).

**FIGURE 3:**
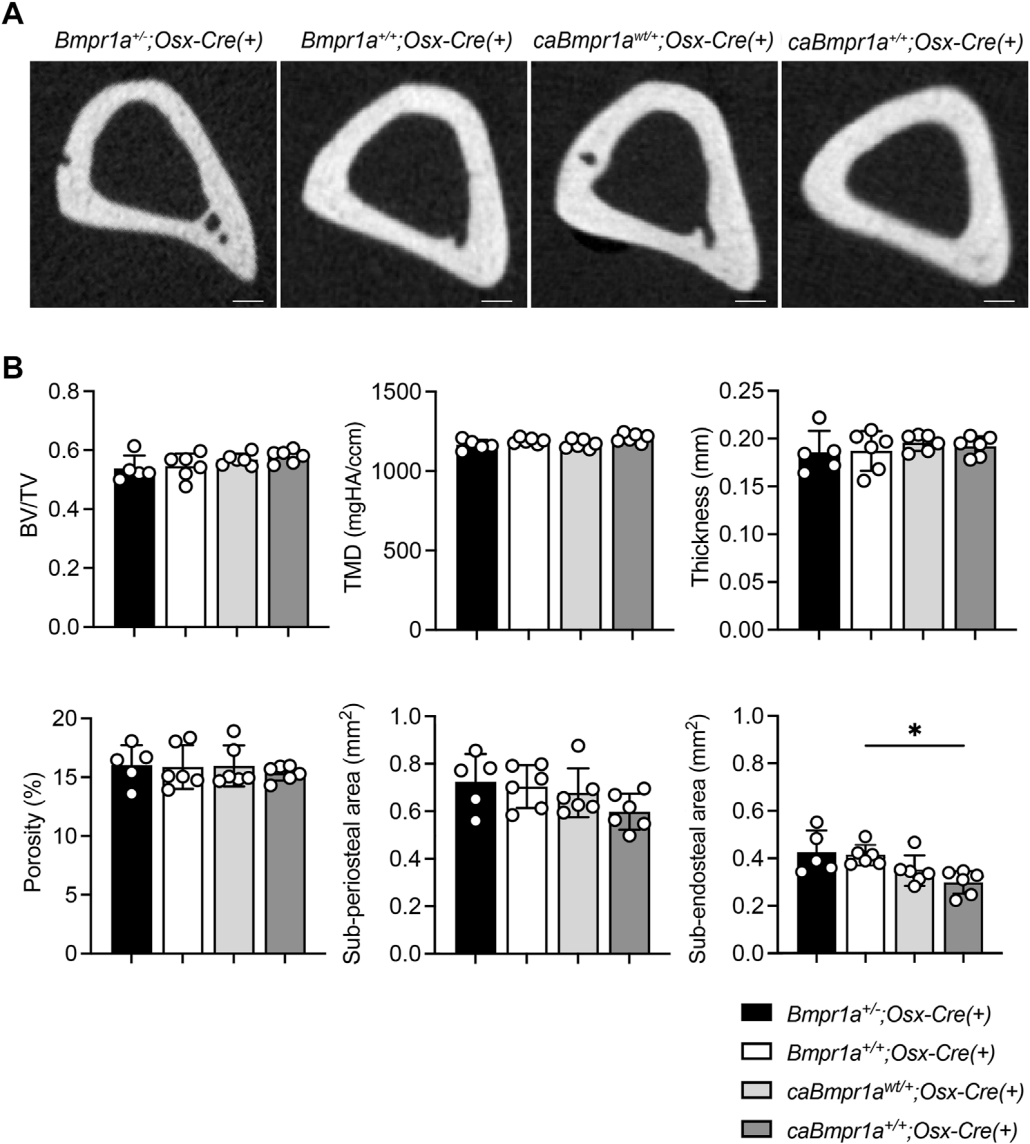
Micro-CT analysis for cortical compartments of the male mouse tibia at 12 weeks of age. **(A)** Micro-CT based 2D images of the mouse proximal tibia. Scale bar = 200 µm. **(B)** Bone volume (BV/TV), tissue mineral density (TMD), thickness, porosity, sub-periosteal (total) area and sub-endosteal (marrow) area were analyzed (n = 5 male for *Bmpr1a* ^*+/−*^ *;Osx-Cre* mice, n = 6 male for *Bmpr1a* ^*+/+*^ *;Osx-Cre, caBmpr1a* ^*wt/+*^ *;Osx-Cre, caBmpr1a* ^*+/+*^ *;Osx-Cre* mice). **p < 0.01.

**FIGURE 4:**
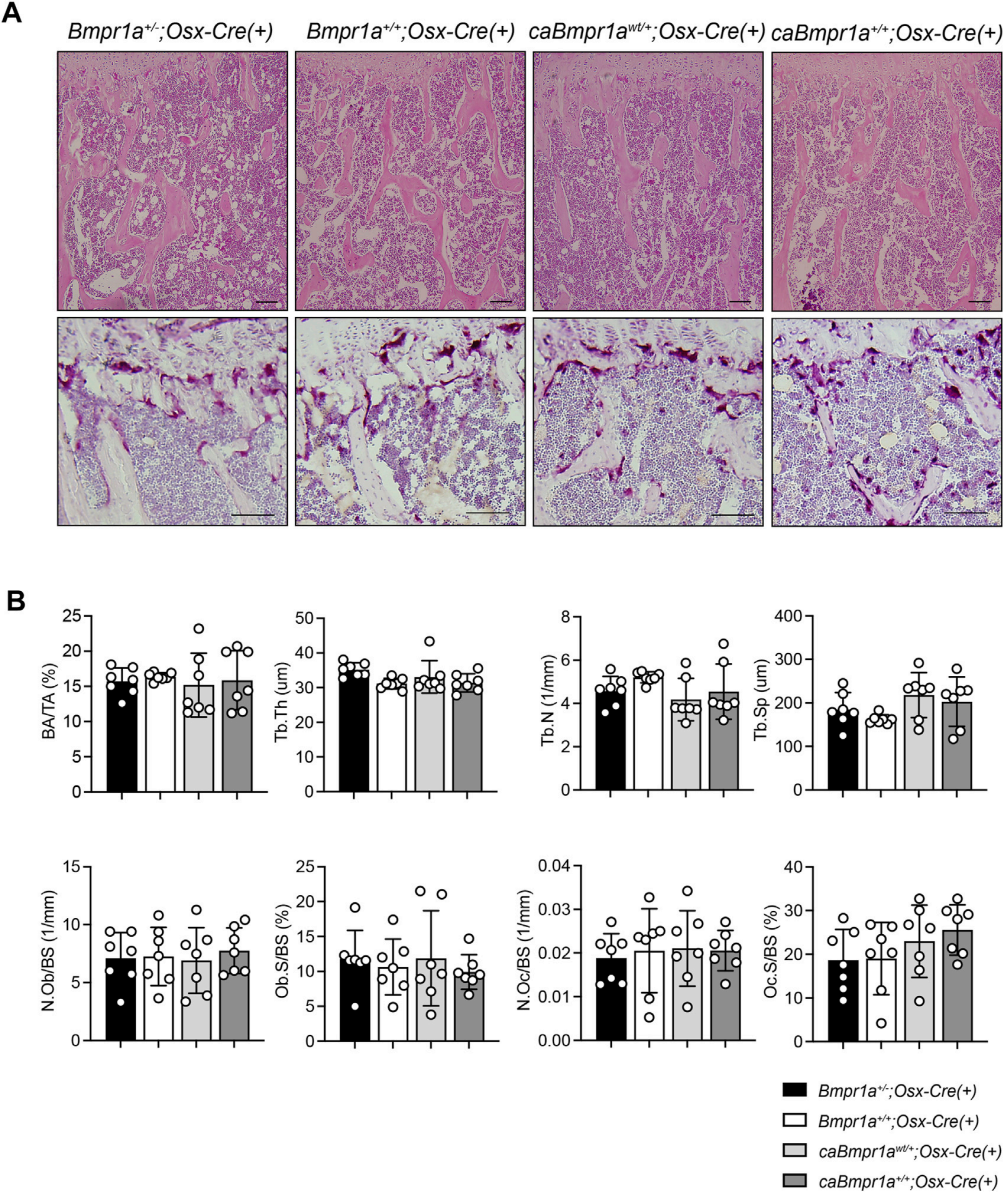
**(A)** Top, H&E staining of the mouse proximal tibia. Scale bar = 100 µm. Bottom, TRAP staining of the mouse proximal tibia. Scale bar = 50 µm. **(B)** Bone area/tissue area (BA/TA), trabecular thickness (Tb.Th), trabecular number (Tb.N), trabecular space (Tb.Sp), osteoblast number/bone surface (N.Ob/BS), osteoblast surface/bone surface (Ob.S/BS), osteoclast number/bone surface (N.Oc/BS), osteoclast surface/bone surface (OcS/BS) were analyzed (*n* = 7 male for each group). ***p < 0.001, **p < 0.01.

Micro-CT analysis for the trabecular compartments of the tibia at 3 months showed no overt differences in BV/TV, BMD, Tb.N, Tb.Th, Tb.Sp and Conn.D among groups (Figures 2A, B). For the cortical compartments of the tibia, there were no overt differences in BV/TV, TMD, thickness, porosity, and sub-periosteal area while sub-endosteal area of the *caBmpr1a* ^*+/+*^ *;Osx-Cre* tibia was smaller than controls (Figures 3A, B). Morphometric assessment of H&E-stained tibiae revealed no overt differences in BA/TA, Tb.N, Tb.Th and Tb.Sp among groups (Figures 4A, B). In terms of osteoblast number (N.Ob/BS), osteoclast number (N.Oc/BS), osteoblast surface (Ob.S/BS) and osteoclast surface (Oc.S/BS), there were no significant differences among groups.

### 3.3 Modest changes in gene expression of bone formation and bone resorption markers in *Bmpr1a* heterozygous null and *caBmpr1a* mutant mice

**FIGURE 5:**
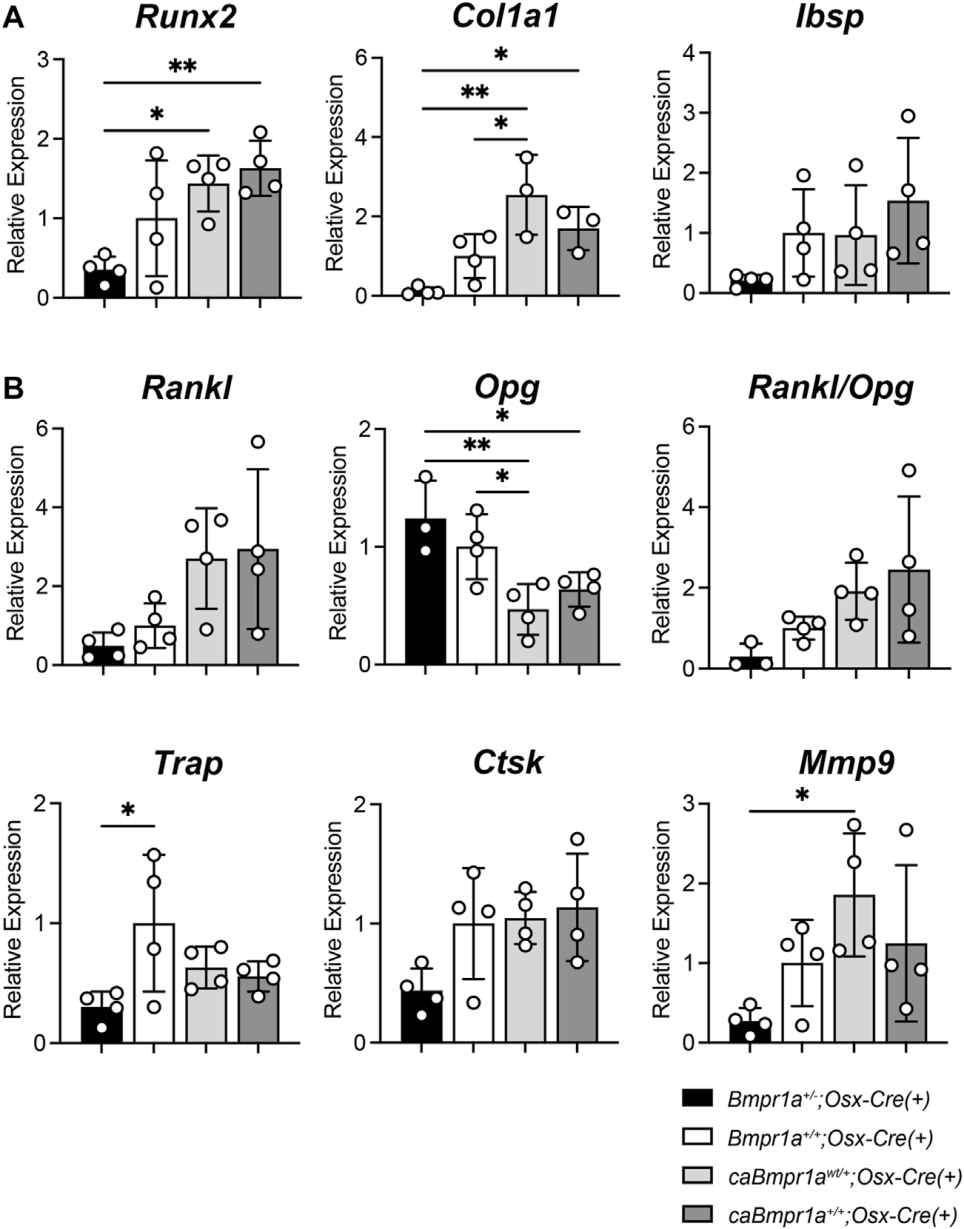
Gene expression for bone formation and bone resorption markers in the male mouse tibia at 12 weeks of age. **(A)** Bone formation marker genes (*Runx2, Col1a1, Ibsp*) were analyzed. (n = 4 each group). **(B)** Bone resorption marker genes (*Rankl, Opg, Trap, Ctsk, Mmp9*) were analyzed (n = 4 male for each group). **p < 0.01 and *p < 0.05.

Quantitative reverse transcribed (RT)-PCR of the tibia showed that the *caBmpr1a* ^*wt/+*^ *;Osx-Cre* and *caBmpr1a* ^*+/+*^ *;Osx-Cre* tibiae exhibited higher expression of bone formation markers such as *Col1a1* and *Runx2* than the *Bmpr1a* ^*+/−*^ *;Osx-Cre* tibiae (Figure 5A). In terms of bone resorption markers, expression levels of *Opg,* a decoy receptor for RANKL which inhibits osteoclastogenesis, were lower in the *caBmpr1a* ^*wt/+*^ *;Osx-Cre* and *caBmpr1a* ^*+/+*^ *;Osx-Cre* mice than the *Bmpr1a* ^*+/−*^ *;Osx-Cre* mice (Figure 5B).

### 3.4 Increased BMP signaling activity in *caBmpr1a* mutant mice in ligand-dependent and ligand-independent manners

**FIGURE 6:**
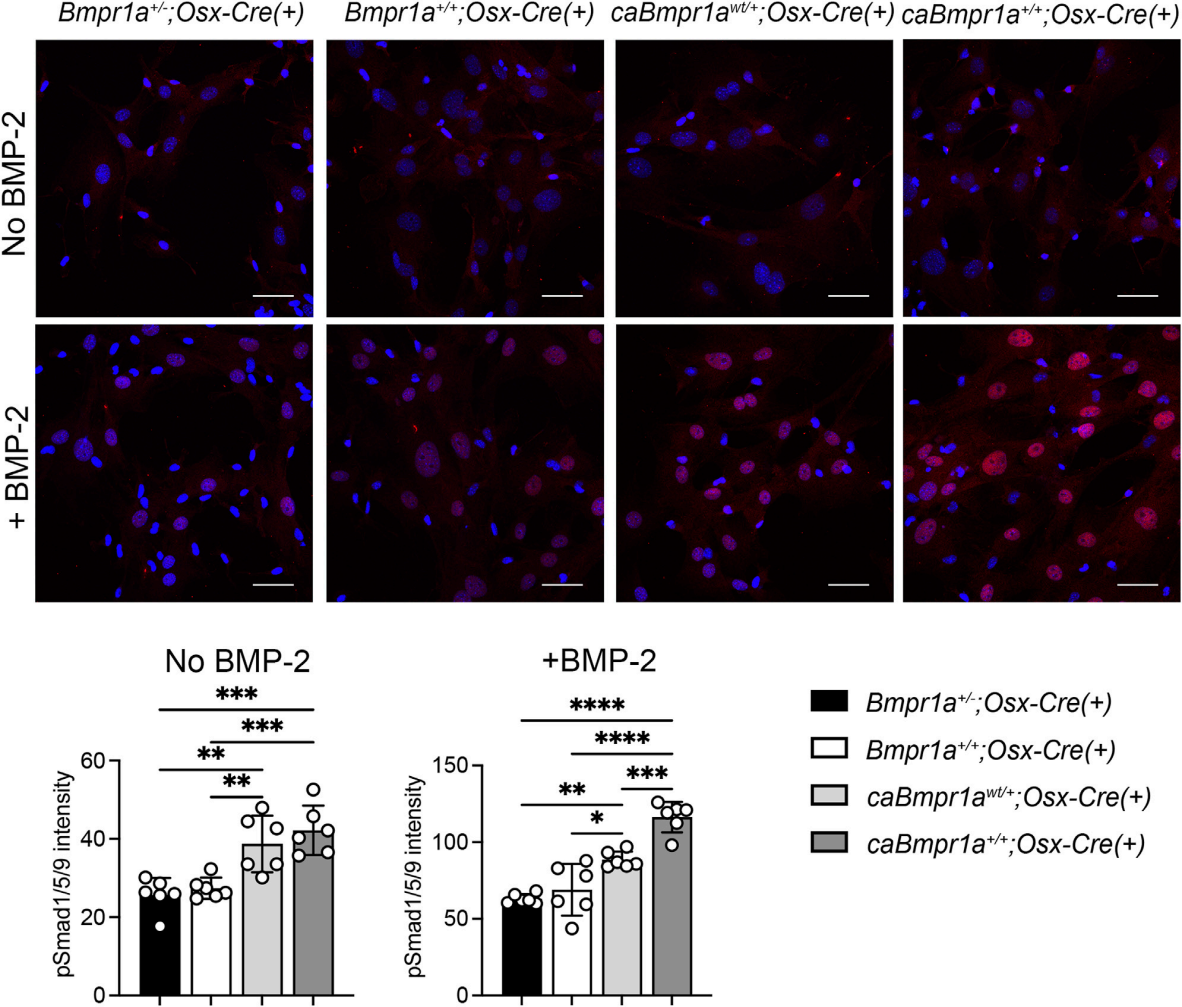
Immunofluorescence detection of phosphorylated form of Smad1/5/9 in bone marrow stromal cells (BMSCs) from the mouse tibia. BMSCs were incubated with/without 100 ng/mL of rhBMP-2 for 30 min. Intensity of pSmad1/5/9 (red) in the nucleus was measured (n = 6 male for each group). Scale bar = 50 μm ****p < 0.0001, ***p < 0.001, and **p < 0.01.

To investigate the BMP signaling activity in each group, phosphorylation levels of Smad1/5/9 (pSmad1/5/9) in bone marrow stromal cells (BMSCs) isolated from tibiae were determined by immunofluorescence intensity of pSmad1/5/9 signal in nucleus after 5 h in culture. BMSCs from the *caBmpr1a* ^*wt/+*^ *;Osx-Cre* and *caBmpr1a* ^*+/+*^ *;Osx-Cre* tibiae exhibited higher pSmad1/5/9 levels than those from the *Bmpr1a* ^*+/−*^ *;Osx-Cre* and *Bmpr1a* ^*+/+*^ *;Osx-Cre* tibiae without BMP-2 stimulation (Figure 6 **)**. With BMP-2 stimulation, BMSCs from the all groups exhibited higher levels of pSmad1/5/9 than those without BMP-2 stimulation. In the presence of BMP-2, BMSCs from the *caBmpr1a* ^*wt/+*^ *;Osx-Cre* and *caBmpr1a* ^*+/+*^ *;Osx-Cre* tibiae exhibited higher levels of pSmad1/5/9 than those from the *Bmpr1a* ^*+/−*^ *;Osx-Cre* and *Bmpr1a* ^*+/+*^ *;Osx-Cre* tibiae.

## 4 Discussion

### 4.1 Overall findings

Upon the discover of BMPs as potent inducers for ectopic bones formation (Urist, 1965), BMPs have been regarded as a golden standard biological means to increase bone mass. However, after over 2 decades of clinical trials and genetic investigations using animal models conducted by our group and others, outcomes of BMP treatment are much more complicated than initially anticipated. We previously demonstrated loss of function of BMP signaling mediated by BMPR1A in osteoblasts results in augmentation of orthotopic bone mass, while osteoblast-specific enhancement of BMPR1A-Smad signaling transgenic mouse line (in which BMPR1A signaling is constitutively activated in early to late osteoblasts) does not cause overt bone phenotypes (Mishina et al., 2004; Kamiya et al., 2008a; Kamiya et al., 2008b; Shi et al., 2018; Kamiya et al., 2020). To gain further insight into the levels of BMP signaling in osteoblasts and bone phenotypes, we took an advantage of the Tet-off *Osx*-*Cre* mouse line to prepare mouse lines with four different levels of BMP-Smad signaling in osteoblasts without necessitating tamoxifen injection. As expected, BMP dependent signal, as denoted by phosphorylated Smad 1/5/9, as well as specific target gene *Id1*, was downregulated in *Bmpr1a* ^*+/−*^ mice and is elevated more in homozygous mice for *caBmpr1a* transgene (*caBmpr1a* ^*+/+*^ *;Osx-Cre* mice) than in heterozygous mice (*caBmpr1a* ^*wt/+*^ *;Osx-Cre* mice). However, it is noted that bone mass was not changed in the homozygous transgenic mice. Bone phenotypes were unchanged regarding trabecular and cortical bone structures and static bone parameters for osteoblasts and osteoclasts. The only change we noticed is a small reduction of sub-endosteal area in homozygous mice for the *caBmpr1a* transgene when compared with *Bmpr1a* ^*+/+*^ mice also carrying *Osx-Cre*. It is noted that both markers for bone formation (*Runx2*, *Col1a1*) and resorption (*Mmp9*, *Opg*, tendency for *Rankl*) were significantly augmented by the upregulated BMP signaling in both hemizygous and homozygous *caBmpr1a* mice, which presumably did not alter the balance of bone metabolic kinetics nor net bone mass. Expression levels of most of the aforementioned genes were not changed between *Bmpr1a* ^*+/−*^ and *Bmpr1a* ^*+/+*^ mice and taken together the facts of no significant changes of levels of pSmad1/5/9 (Figure 1B), *Id1* expression (Figure 1C), and response to BMP-2 in culture (Figure 6), these suggest that one copy of *Bmpr1a* is enough to transduce enough levels of BMP-Smad signaling in *Osterix*-expressing cells. These data suggest that a small upregulation of BMP-Smad signaling in *Osterix*-expressing cells does not alter bone phenotypes and also suggest experimental and clinical outcomes of increased bone mass by BMP treatment is due to its impact on other types of cells such as mesenchymal stem cells.

We previously reported that heterozygous conditional mutations of *caBmpr1a* using 3.2-kb Col1-CreER™ mice results in no overt changes in net bone mass with modest changes in osteoblast and osteoclast activities at 34 weeks of age (Kamiya et al., 2020). In this study, we used *Osterix*-Cre (*Osx*-Cre) transgenic mice which are widely used to target immature to mature osteoblasts; they can generate GFP/Cre fusion protein under the control of the Osterix (Sp7) promoter along with a tetracycline responsive element (Rodda and McMahon, 2006). One advantage of use of *Osx*-Cre is to avoid use of tamoxifen, which may affect bone phenotypes (Broulik, 2000). Because we and others reported that *Osx*-Cre mice without any floxed regions show some bone phenotypes including a cortical bone phenotype and minor craniofacial defects (Razidlo et al., 2010; Davey et al., 2012; Wang et al., 2015), we used mice carrying *Osx*-Cre but wildtype for *Bmpr1a* as controls to compare bone phenotypes and molecular changes.

Unlike an early-stage embryonic lethality caused by conventional homozygous deletion of *Bmpr1a* (Mishina et al., 1995), we have not noticed developmental defects in heterozygous mutant mice (*Bmpr1a* ^*+/−*^). In the adult stage, some of the *Bmpr1a* heterozygous null mice showed an abnormality in glucose metabolism such as higher glucose response and lower insulin levels in the heterozygous mice (Scott et al., 2009); however, the mutation did not cause overt bone abnormalities as reported here. There is a formal possibility that the absence of bone phenotypes in *Bmpr1a* ^*+/−*^ mice may be due to reduced BMPR1A-Smad signaling in other types of cells, because this is a global knockout. Although less likely, this possibility can be addressed by using of a conditional allele of *Bmpr1a* (Mishina et al., 2002), which we previously generated, in combination with *Osx*-Cre.

One of the limitations of the current study is that only male mice were used to limit possible confounding effects of sex hormones in female mice. We previously reported that heterozygous conditional mutations of *caBmpr1a* using 3.2-kb Col1-CreER™ mice resulted in no overt changes in net bone mass both males and females (Kamiya et al., 2020). Thus, we expect to see no overt changes in net bone mass in female mice. Although less likely, there is a formal possibility that mice used in this study may demonstrate different bone phenotypes when they age. In the previous study, we analyzed bone phenotypes at 34 weeks after birth (Kamiya et al., 2020), of which phenotypes are similar with these at 12 weeks old. For the cases of loss-of-function studies, when we delete *Bmpr1a* in an osteoblast-specific manner using *Osteocalcin*-Cre, the mutant mice showed age-dependent outcomes, i.e., lower bone mass at 1 month of age, and higher bone mass at 10 months than littermate controls (Mishina et al., 2004). In contrast, when we used 3.2-kb Col1-CreER™, the mutant mice consistently showed higher bone mass as late-stage embryos, at weaning stages and at 22 weeks old after birth (Kamiya et al., 2008a; Kamiya et al., 2008b; Kamiya and Mishina, 2011). However, future studies would be needed to determine the gender dependence using different age groups. Another limitation may be associated with low sample size (n = 4 to 7 per genotype for microCT analyses). For the animal experiments, the number of mice in each group was determined according to our previous reports (Kamiya et al., 2020). In this study, we observed statistically significant changes in BMP dependent signal, as denoted by pSmad1/5/9 levels, as well as specific target gene *Id1* expression levels in both heterozygous and homozygous mice for *caBmpr1a* although these mice displayed no overt bone phenotypes. Thus, we expect that increases in the sample size do not affect the overall conclusion of this study. However, larger sample sizes may be needed to detect subtle differences among groups.

### 4.2 Clinical aspects

In the clinic, a high dose of BMP-2, such as 12 mg in a concentration of 1.5 mg/mL (FDA, 2002), has been used for fracture repair to induce a bone formation in patients expecting it functions through osteoblasts. However, such high doses of BMPs may introduce unexpected adverse effects (i.e., bone resorption, inflammation, ectopic ossification), likely due to interacting with other types of cells rather than osteoblastic cells (Kim et al., 2013). It is assumed that such clinical side effects are caused by the effects of high dose BMPs on non-bone tissues. Thus, the dosage administered and the way to distribute BMPs are important to be considered for better treatment of BMP-2 therapy. A dose-response with exogenous BMPs would be desired to investigate the multifaceted functions of BMPs *in vivo*. It is noted that BMPs can directly control cartilage formation to positively affect endochondral bone formation by increasing the size of bone templates (Kamiya, 2012; Zhang et al., 2022).

Recently several lines of evidence for BMP-6 as an alternative treatment for orthopedic conditions have been accumulated. BMP-6 is superior to BMP-2 and BMP-7 in its activity to stimulate bone formation *in vitro* and *in vivo* (Vukicevic and Grgurevic, 2009; Song et al., 2010) because it can activate all three type I receptors for BMPs. Additionally, unlike BMP-2 and BMP-7, BMP-6 is resistant to Noggin, a major BMP antagonist found in bones (Song et al., 2010), allowing the use of low BMP-6 concentration with autologous blood coagulum (ABC) (Sampath and Vukicevic, 2020). In human, an autologous bone graft substrate (ABGS), an improved version of ABC (Grgurevic et al., 2019), has been tested for patients with distal radial fracture (Phase I) (Durdevic et al., 2020), and for patients receiving high tibial osteotomy (Phase I/II) (Chiari et al., 2020). In this therapy, ABGS containing 250 μg rhBMP-6 per mL into the fracture site between two ends and was proven safe and efficacious. This is a highly promising avenue for human applications due to the reduced BMP concentration that can reduce adverse reactions.

## 5 Conclusion

In this study, we bred several lines of mutant mouse lines such as conventional knockout allele of *Bmpr1a* and the constitutively activated BMPR1A allele to generate mice with 4 different doses of BMP-Smad signaling in early to late osteoblasts and investigated the impact of different levels of BMP signaling on endogenous long bones in adults. While alterations in expression levels of bone formation and resorption markers were noted at transcriptional levels, the net bone mass was unchanged in the mutant mice. This study clearly demonstrated a discrepancy between physiological functions of BMP-Smad signaling and expected outcomes in the clinical setting, which provides a new insight in considering a better and more efficient therapeutic regime to mitigate potential side effects by using high dose of BMPs.

## Data Availability

The original contributions presented in the study are included in the article/Supplementary Materials, further inquiries can be directed to the corresponding author.
