# Supplementary material for: Increased BMP-Smad signaling does not affect net bone mass in long bones
Source: Front Physiol. 2023 Mar 30;14:1145763. doi: 10.3389/fphys.2023.1145763 (PMC10101206; doi:10.3389/fphys.2023.1145763)
Supplement: Supplementary file 1 [file Table1.DOCX]

Table. S1. Sequences of primers used in quantitative real-time polymerase chain reaction for gene expression analysis.

| Gene | Forward | Reverse |
| --- | --- | --- |
| Col1a1 | GCTCCTCTTAGGGGCCACT | CCACGTCTCACCATTGGGG |
| IBSP | CAGGGAGGCAGTGACTCTTC | AGTGTGGAAAGTGTGGCGTT |
| RUNX2 | TGGCCGGGAATGATGAGAAC | TGAAACTCTTGCCTCGTCCG |
| GAPDH | CGTCCCGTAGACAAAATGGT | TTGATGGCAACAATCTCCAC |
| RANKL | GCACACCTCACCATCAATGCT | GGTACCAAGAGGACAGAGTGACTTTA |
| Opg | ACCCAGAAACTGGTCATCAGC | CTGCAATACACACACTCATCACT |
| Acp5 (TRAP) | CGTCTCTGCACAGATTGCA | GAGTTGCCACACAGCATCAC |
| Ctsk | AGGGAAGCAAGCACTGGATA | GCTGGCTGGAATCACATCTT |
| Mmp9 | CGCTCATGTACCCGCTGTAT | CCGTGGGAGGTATAGTGGGA |
